# Origin and distribution of epipolythiodioxopiperazine (ETP) gene clusters in filamentous ascomycetes

**DOI:** 10.1186/1471-2148-7-174

**Published:** 2007-09-26

**Authors:** Nicola J Patron, Ross F Waller, Anton J Cozijnsen, David C Straney, Donald M Gardiner, William C Nierman, Barbara J Howlett

**Affiliations:** 1 Department of Botany, University of British Columbia, Vancouver, British Columbia, V6T 1Z4, Canada; 2 School of Botany, the University of Melbourne, Victoria 3010, Australia; 3 Department of Cell Biology and Molecular Genetics, University of Maryland, College Park, MD 20742, USA; 4 CSIRO Plant Industry, 306 Carmody Rd, St Lucia, QLD 4072, Australia; 5 J. Craig Venter Institute, 9704 Medical Center Drive, Rockville, MD 20850 USA, and The George Washington University School of Medicine, Department of Biochemistry and Molecular Biology, Washington, DC 20037, USA

## Abstract

**Background:**

Genes responsible for biosynthesis of fungal secondary metabolites are usually tightly clustered in the genome and co-regulated with metabolite production. Epipolythiodioxopiperazines (ETPs) are a class of secondary metabolite toxins produced by disparate ascomycete fungi and implicated in several animal and plant diseases. Gene clusters responsible for their production have previously been defined in only two fungi. Fungal genome sequence data have been surveyed for the presence of putative ETP clusters and cluster data have been generated from several fungal taxa where genome sequences are not available. Phylogenetic analysis of cluster genes has been used to investigate the assembly and heredity of these gene clusters.

**Results:**

Putative ETP gene clusters are present in 14 ascomycete taxa, but absent in numerous other ascomycetes examined. These clusters are discontinuously distributed in ascomycete lineages. Gene content is not absolutely fixed, however, common genes are identified and phylogenies of six of these are separately inferred. In each phylogeny almost all cluster genes form monophyletic clades with non-cluster fungal paralogues being the nearest outgroups. This relatedness of cluster genes suggests that a progenitor ETP gene cluster assembled within an ancestral taxon. Within each of the cluster clades, the cluster genes group together in consistent subclades, however, these relationships do not always reflect the phylogeny of ascomycetes. Micro-synteny of several of the genes within the clusters provides further support for these subclades.

**Conclusion:**

ETP gene clusters appear to have a single origin and have been inherited relatively intact rather than assembling independently in the different ascomycete lineages. This progenitor cluster has given rise to a small number of distinct phylogenetic classes of clusters that are represented in a discontinuous pattern throughout ascomycetes. The disjunct heredity of these clusters is discussed with consideration to multiple instances of independent cluster loss and lateral transfer of gene clusters between lineages.

## Background

**Figure 1:**
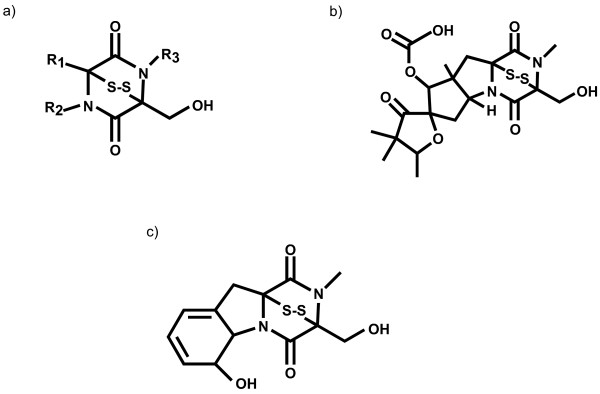
Structures of a/the core moiety of an epipolythiodioxopiperazine (ETP); b/sirodesmin PL; c/gliotoxin.

Filamentous fungi produce a diverse array of secondary metabolites. These include polyketides (e.g. aflatoxins), cyclic peptides, alkaloids and sesquiterpenoids (e.g. trichothecenes)[1]. Epipolythiodioxopiperazines (ETPs) are a poorly studied class of secondary metabolite toxins derived from cyclic peptides [2]. These toxins are made by a phylogenetically diverse range of filamentous fungi and are characterised by the presence of a disulphide bridge (Fig. 1) that allows ETPs to cross-link proteins via cysteine residues and to generate reactive oxygen species through redox cycling, properties that confer toxicity. Epipolythiodioxopiperazines are implicated in several animal and plant diseases, however, their distribution in fungi is poorly understood. The role of ETPs within the fungus is unknown, although these molecules may confer an advantage to the fungus growing within its ecological niche.

Genes responsible for biosynthesis of fungal secondary metabolites are usually tightly clustered in the genome and co-regulated in a manner consistent with the timing of production of the metabolite. Furthermore if steps in the biosynthetic pathway can be deduced, the gene cluster can often be identified by searching genome sequences for linked genes encoding appropriate enzymes [1].

**Figure 2:**
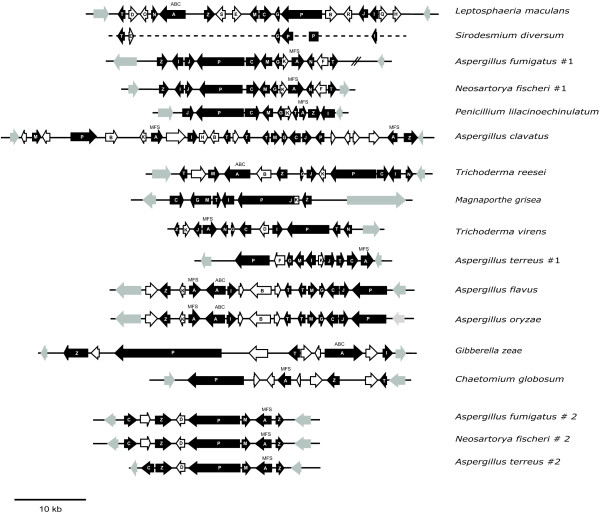
**Putative ETP biosynthetic gene clusters in ascomycetes**. Genes (white text on black background) include those with best matches to non-ribosomal peptide synthetase (*P*), thioredoxin reductase (*T*), methyl transferases (*M *and *N*), glutathione *S*-transferase (*G*) and cytochrome P450 monooxygenase (*C*), ACCS (*I*), dipeptidase (*J*), as well as a transcriptional regulator (*Z*) and a transporter (*A *– Multi Facilitator Superfamily (MFS) or ABC). In addition genes with predicted roles in modification of the side chains of the core ETP moiety are noted (see Table 1). Genes with no lettering are either hypothetical or have no strong matches to ETP cluster genes. Genes shaded in grey are predicted to flank the cluster and encode proteins with best matches to proteins with no potential roles in ETP biosynthesis. The cluster in *T. virens *might be incomplete as a gene common to ETP cluster was at one end of a single cosmid clone. In *M. grisea G *and *M*, and *P*, *J *and *K *are annotated as fused genes. The arrangement of genes in *S. diversum *is based on the sirodesmin PL cluster in *L. maculans*; the dashed line represents unsequenced regions. '#2' after taxon name indicates that this is the second cluster found in that species.

The gene clusters responsible for biosynthesis of ETP toxins sirodesmin PL and gliotoxin in the ascomycetes *Leptosphaeria maculans *and *Aspergillus fumigatus*, respectively, have been identified [3,4]. They are 55 and 28 kb in length, respectively and ten genes are common to both (Fig. 2). Eight of these genes encode enzymes with high similarity to a two module non-ribosomal peptide synthetase (*P*); thioredoxin reductase (*T*); O-methyl transferase (*M*); a methyl transferase with unknown specificity (*N*); glutathione *S*-transferase (*G*); cytochrome P450 monooxygenases (*C*); amino cyclopropane carboxylate synthase (ACCS) (*I*); and a dipeptidase (*J*). While the role of a few of these genes can be deduced (for example; *P*, which catalyses the condensation of two amino acids in the initial biosynthetic step [5]), for the others their role in ETP biosynthesis is unknown. Two other genes encode common functions in both clusters, a zinc binuclear (Zn(II)_2_Cys_6_) transcriptional regulator (*Z*) that controls expression of the biosynthetic enzymes [6], and a transporter (*A*) involved in toxin efflux [7]. Additional genes are predicted to encode enzymes involved in the modification of the core ETP moiety, however, no functional analyses of genes other than the peptide synthetase (*P*) and the transcriptional regulator (*Z*) have been reported (Table 1). The expression of genes in these clusters in *L. maculans *and *A. fumigatus *are co-regulated consistent with the timing of production of the respective ETP [3,4]. Further, when the peptide synthetases (*P*) in these clusters are disrupted, the resultant mutants are unable to produce sirodesmin PL and gliotoxin, respectively, consistent with the clusters encoding the biosynthetic enzymes [3,8]. Recent studies of these mutants, and also a mutant in the transcriptional regulator (Z) in *A. fumigatus *have shown that gliotoxin contributes to virulence of *A. fumigatus *in immunocompromised mice and that sirodesmin PL contributes to virulence in *L. maculans *in the plant *Brassica napus *[6,8,9].

The genome of the rice blast fungus, *Magnaporthe grisea*, has a cluster with nine common genes and the wheat head scab fungus, *Gibberella zeae *(anamorph *Fusarium graminaerum*)has a cluster containing five common genes [3]. However, ETP production by these fungi has not been recorded. Gliotoxin production by some distantly related ascomycetes (for example, *Aspergillus fumigatus, Trichoderma virens *and *Penicillium terlikowskii *has been reported [4,10,11]. Also other *Aspergillus *species such as *A. flavus*, *A. niger *and *A. terreus *produce gliotoxin [12]. It is intriguing that this trait (gliotoxin production) has achieved such a disjunct distribution across ascomycetes. Complete fungal genome sequences are now available for many fungi, thus allowing the distribution of potential ETP gene clusters across a range of species.

In this paper we describe the distribution of ETP-like gene clusters in a range of ascomycetes and the analyses of phylogenetic relationships of several cluster genes and their paralogues throughout the ascomycota. These relationships have been used to examine the origin of ETP gene clusters, and the pattern of heredity of the cluster constituents. These data are used to address whether these putative ETP gene clusters formed independently in the various lineages of the ascomycota. Phylogenies inferred by cluster proteins have been used to assess the likelihood of two processes of cluster inheritance within ascomycota: vertical inheritance coupled with multiple cluster loss, and horizontal (lateral) transfer of entire clusters.

## Results

### Identification and characteristics of putative ETP biosynthetic gene clusters

Putative ETP biosynthetic gene clusters identified from a range of filamentous ascomycetes are presented in Figure 2. These clusters were identified by comparative analyses of genomic data of varying states of sequence coverage and annotation [see Additional file 1]. Also clusters were cloned from gliotoxin-producing isolates of *Trichoderma virens *and *Penicillium lilacinoechinulatum *(the strain of this latter fungus had previously been wrongly identified as *Penicillium terlikowskii *– see Methods section), for which no genomic data exist. Cosmid libraries of these two fungi were screened with a homologue of ACCS (*I*) and cosmids that hybridised, and had several of the common ETP cluster genes, were sequenced and analysed. Using primers based on genes in the sirodesmin PL and the gliotoxin clusters, part of a putative ETP cluster was also obtained from *Sirodesmium diversum*, a fungus that produces sirodesmin, although a different chiral isomer to that produced by *L. maculans *[13,14]. The order of genes in this putative cluster is based on that of the sirodesmin PL cluster in *L. maculans*. Throughout the fungi examined, putative ETP gene clusters were identified by the presence of linked genes with best matches to eight of the common ETP cluster genes (*P*, *T*, *M*, *N*, *G*, *C*, *I *and *J*) previously described from *L. maculans *and *A. fumigatus*. The cluster in *T. virens *might be incomplete as a gene common to ETP cluster was at one end of a single cosmid clone; further linked genes may be part of this cluster.

Putative ETP clusters, containing strong matches to eight of the genes (*P*, *T*, *M*, *N*, *G*, *C*, *I *and *J*) common to the clusters in *L. maculans *and *A. fumigatus *were identified in *Neosartorya fischeri, P. lilacinoechinulatum *and *Trichoderma reesei*, as well as in *M. grisea*. Several other fungi had clusters missing one of these genes. Clusters in *A. flavus, A. oryzae, A. terreus *and *A. clavatus *lacked the gene encoding a methyl transferase (*N*). *Trichoderma virens *lacked an *O*-methyl transferase (*M*), although it had an extra methyl transferase (*N*). The non-ribosomal peptide synthetases (*P*) in all the clusters were bimodular, consisting of two peptide synthetase units in tandem, except for that in *G. zeae*, which had four modules. Curiously, in the *A. clavatus *cluster the gene for peptide synthetase (*P*) has apparently split into two separate coding sequences (although not equally separating the two modules) that are convergently transcribed (Fig. 2). Despite rearrangement, the predicted coding sequence of these two genes added together shares high sequence identity with that of the single *P *from *A. oryzae *and *A. flavus*.

The gliotoxin cluster of *A. fumigatus *had a cytochrome P450 monooxygenase (*F*) with low sequence similarity to *C *and a hypothetical gene (*K*). These two genes are not present in the sirodesmin PL gene cluster [3]. The hypothetical gene (*K*) was present only in clusters from fungi known to produce gliotoxin (*P. lilacinoechinulatum, T. virens, A. terreus, A. flavus *and *A. oryzae). Aspergillus terreus *also had the cytochrome P450 monooxygenase (*F*). Other putative ETP clusters had additional genes or even several copies of one particular gene; for instance, the clusters in *A. flavus *and *A. oryzae *had two thioredoxin reductases (*T*), whilst *A. clavatus *had three copies (Fig. 2).

The zinc binuclear (Zn(II)_2_Cys_6_) transcriptional regulator (*Z*) was present in all clusters except in the larger cluster (cluster #1) of *A. terreus *(Fig. 2). Transporters (*A*) of either ATP Binding Cassette (*ABC*) or Multi Facilitator Superfamily (*MFS*) type were present in all clusters except *M. grisea. Aspergillus flavus *and *A. oryzae *had two adjacent transporter genes – one an ABC and the other an MFS type.

Two taxa, *G. zeae *and *Chaetomium globosum*, had clusters with fewer of the common genes. A non-ribosomal peptide synthetase (*P*), a thioredoxin reductase (*T*), a transcriptional regulator (*Z*) and a transporter (*A*) occurred in both, and in the case of *G. zeae*, an ACCS (*I*) also (Fig. 2). Other genes in the *G. zeae *cluster had best matches to two different hydrolases, an esterase and a gene with some but not all of the domains of a single module of a peptide synthetase. Other genes in the *C. globosum *cluster included ones with best matches to genes with potential roles in secondary metabolism – a cytochrome P450 monooxygenase (with little sequence similarity to C), a methyl transferase (neither of which had strong matches to those in ETP clusters) as well as a hypothetical protein and an enoyl reductase. Clusters bearing a reduced set of common genes were also found as second clusters in *A. fumigatus, N. fischeri *and *A. terreus *(Fig. 2, denoted #2). These all contained *M, P *and *C*, as well as a transporter (*A*) and two transcriptional regulators (*Z*). This cluster in *A. fumigatus *and *N. fischeri *also had a gene with a Flavin Adenine Dinucleotide (FAD) binding domain between *Z *and *C*; this gene was not present in the *A. terreus *cluster.

Analysis of complete genome sequences of a range of ascomycetes (*Saccharomyces cerevisiae*, *Schizosaccharomyces pombe*, *Neurospora crassa*, *Ajellomyces capsulatus*, *Ascophaeria apis*, *Aspergillus nidulans*, *Botryotinia fuckleliana*, *Coccidioides immitis*, *Phaeosphaeria nodorum*, *Uncinocarpus reesii*), as well as the basidiomycetes (*Ustilago maydis *and *Cryptococcus neoformans*) did not reveal any putative ETP gene clusters in these taxa.

The putative ETP clusters typically occurred in different genomic locations in each lineage analysed. For instance, even between closely related fungi such as *N. fischeri *and *A. fumigatus *where within the larger cluster gene order was otherwise identical and sequence was extremely similar, the genes flanking these clusters were different.

### Most ETP clusters have a single origin

In order to infer phylogenies of the ETP cluster components, the deduced amino acid sequences for all the common cluster genes, except the transporter (*A*) and the transcriptional regulator (*Z*), were aligned with homologues throughout the ascomycetes as well as from the basidiomycetes *U. maydis *and *C. neoformans *as outgroup taxa. Six of these proteins contained sufficient alignable characters for phylogenetic analysis. These were the two-module non-ribosomal peptide synthetase (P), cytochrome P450 monooxygenase (C), glutathione S-transferase (G), amino cyclopropane carboxylate synthase (I), dipeptidase (J), and methyl transferase (M). Of these six genes, the class with the largest number of paralogues in the genome was cytochrome P450 monooxygenase. These genes have varying degrees of sequence similarity to each other and are involved in a myriad of cellular functions [15]. For each of the six proteins a preliminary phylogeny was constructed from large alignments containing all available homologues. Based on this analysis, proteins encoded by the more distally related genes to the cluster genes were excluded, allowing the use of a larger character inclusion set for each subsequent analysis.

**Figure 3:**
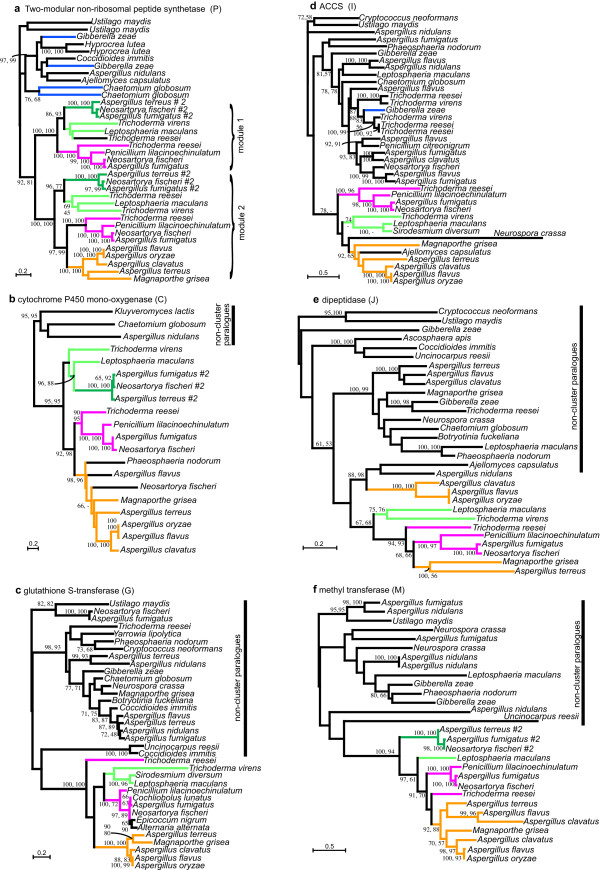
**Phylogenetic relationships between individual proteins encoded in ETP gene clusters**. (a) Two module non-ribosomal peptide synthetase P; (b) cytochrome P450 monooxygenase C; (c) glutathione *S*-transferase G; (d) ACCS I; (e), dipeptidase J; (f) O-methyl transferase M. Numbers at nodes are bootstrap support values from (left to right or top to bottom) PhyML and WEIGHBOR. Proteins encoded within clusters are on coloured branches, and non-cluster paralogues are on black branches. Consistent cluster relationships across the six proteins are indicated by orange (subclade I), pink (subclade II) and light and dark green (subclades IIIA and IIIB, respectively). Proteins encoded in clusters outside of the main cluster clade are on blue branches. '#2' after taxon name indicates that this is the second cluster found in that species, as per Figure 2.

For each of the six protein phylogenies the majority of the cluster proteins formed a well-supported clade (ML bootstraps for cluster clades were: P, 92; C, 95; G, 100; I, 78; J, 67; M, 100) to the exclusion of the non-cluster paralogues (Fig. 3). The size of the character inclusion sets used differed for each protein (see methods), the largest being from the peptide synthetase, P, which accordingly gave the most robust topology through this phylogeny. However, the phylogenetic relationships based on each of the six proteins were consistent, broadly supporting the monophyly of cluster genes. The only exceptions were firstly the cluster genes from *G. zeae *(P and I) and *C. globosum *(P); and secondly the J proteins from *A. clavatus, A. flavus *and *A. oryzae*; all of which nested within the non-cluster paralogues.

For the bimodular non-ribosomal peptide synthetases (P), only the most closely related paralogues of this protein that did not compromise the inclusion set were analysed. The predicted protein sequence was split into the two modules (1 and 2), and all individual modules were aligned, with the exception of module 1 of proteins encoded in the clusters in *A. flavus*, *A. oryzae*, *A. terreus *and *M. grisea*, which were unusually divergent in these taxa. Within the strongly supported cluster clade of P modules, module 1 formed its own subclade, as did module 2. Moreover, modules 1 and 2 were firmly supported as one another's sister clades.

A few proteins not encoded by genes in clusters grouped within the cluster clade: for instance, a C from *P. nodorum *and an I from *Ajell. capsulatus*. These two fungi have fully sequenced genomes and have no ETP-like gene clusters. Glutathione *S*-transferases (G) in *Epicoccum nigrum *and *Alternaria alternata *also grouped within the cluster clade. Also a divergent I from *N. crassa*, a taxon that has no ETP-like clusters, was on a long branch within the cluster clade. The position of this protein may be an artefact, however, tests for long-branch attraction were inconclusive (data not shown). Regardless, removal of this sequence did not affect the topology of the cluster clade nor decrease support for it. In addition to genes from taxa without ETP-like gene clusters, some taxa with clusters contained non-cluster paralogues grouping within the cluster clade. These included a non-cluster cytochrome P450 monooxygenase (C) from *A. flavus *and *N. fischeri*, and a non-cluster P from *T. reesei *(Fig. 3).

### Gene clusters have consistent patterns of inheritance

In addition to cluster genes consistently grouping together in phylogenies, in most cases the relationships within the cluster clade were also consistent for the six individual proteins. The internal topology of the cluster clade generally resolved four subclades – I (orange), II (pink), IIIA (light green), IIIB (dark green) (Fig 3).

Subclade I (orange) included proteins from *M. grisea, A. terreus, A. flavus, A. oryzae *and *A. clavatus*. Support for this subclade was strong for all proteins (ML bootstraps were 100, 98, 100, 92, 92 for P (module 2), C, G, I and M, respectively) except for J (see below). In the phylogeny based on C, the three non-cluster paralogues from *P. nodorum*, *A. flavus *and *N. fischeri *also grouped within subclade I.

Subclade II (pink) included proteins from *T. reesei, P. lilacinoechinulatum, A. fumigatus *and *N. fischeri*. This subclade resolved with strong support for P (both modules), C and I, (ML 100, 90, 100 in Figs. 3a, b and 3d, respectively). In the phylogenies inferred by G, J and M, the position of *T. reesei *was unresolved, most likely due to the small sizes of the inclusion sets derived from these relatively small proteins. The inclusion of more taxa might provide more resolution to these relationships. Subclades I and II were recovered as sisters with good support by four of the proteins (ML 97, 92, 94, 91 in P, C, J and M respectively). In the other two phylogenies (of G and I) the internal branches of the cluster clade were unresolved.

Two further cluster subclades were designated IIIA (light green) and IIIB (dark green). Subclade IIIA included proteins encoded in the *L. maculans *sirodesmin PL cluster [3], as well as the *T. virens *cluster. Additionally, two genomic fragments (encoding *I *and *G*) from *S. diversum *also grouped in this cluster type. The fragments of *P *for this species were too short to be included in the phylogenetic analysis, however, their nucleotide sequences had a high degree of similarity to that of the *L. maculans P *(data not shown). The support for this subclade is relatively weak especially where the *S. diversum *gene fragments, which are all short, are included. Full-length sequences and better representation is needed for resolution of the position of this subclade. Nevertheless, the relationship of the cluster in the sordariomycete *T. virens *to that in the sirodesmin-producing dothideomycetes is consistent throughout the phylogenies (Fig. 3).

Subclade IIIB (dark green) consisted of proteins from the second smaller cluster (cluster #2) of *A. fumigatus*, *N. fischeri *and *A. terreus *(Fig. 2). These proteins were all highly similar, forming a very robust subclade (Figs. 3a, b and 3f). Although only three proteins from these clusters could be analysed, in all cases subclade IIIB is allied to subclade IIIA with weak to moderate support, with greatest support seen in the P phylogeny (Fig. 3).

**Figure 4:**
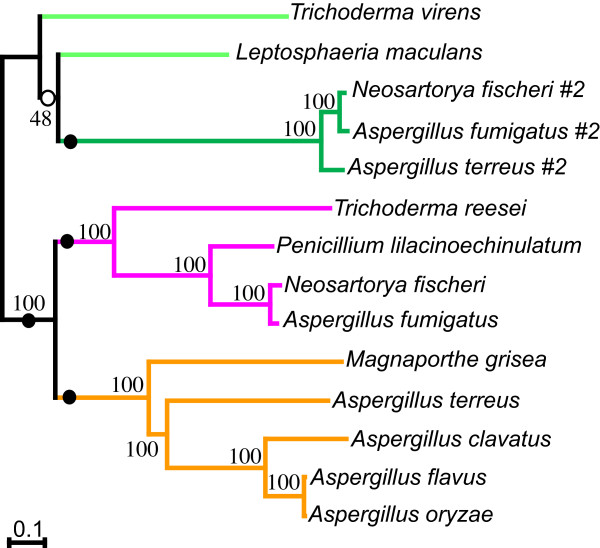
**Maximum likelihood phylogeny inferred from six concatenated proteins encoded in ETP gene clusters**. Numbers at nodes are bootstrap supports obtained from PhyML. Filled circles indicate that Approximate Unbiased (AU) tests rejected alternate topologies that disrupt these nodes (p < 0.003 in all cases). Open circle indicates that disruption of this node was not rejected. '#2' after taxon name indicates that this is the second cluster found in that species, as per Figure 2.

The relationships of the clusters were further tested by analysis of concatenated protein sequences. With the exception of J from *A. flavus*, *A. clavatus *and *A. oryzae *(Fig. 3e) and those encoded by *G. zeae *and *C. globosum *(blue branches in Fig. 3), the proteins encoded in the clusters show a common origin from a single ancestral cluster. Hence, these clusters can be considered homologous, and thus only these proteins were concatenated to form a 'super-matrix'. The maximum likelihood phylogeny inferred from this dataset was strongly supported at almost all nodes, indicating that this larger data set enables much greater resolution than that for each protein alone (Fig. 4). Only support for subclade IIIA was weak. Approximate unbiased tests of alternate tree-topologies rejected (p < 0.003 in all cases) any tree that broke the monophyly of subclade I (orange), subclade II (pink) or subclade IIIB (dark green) (Fig 4). Further, trees where subclades I and II were not sisters were also rejected. This analysis strongly supports the occurrence of distinct cluster subclades, and that subclade I and II are most closely related. It also indicates that the monophyly of cluster IIIA and the relatedness of clusters IIIA and IIIB is uncertain.

### The distribution of ETP clusters is discontinuous

**Figure 5:**
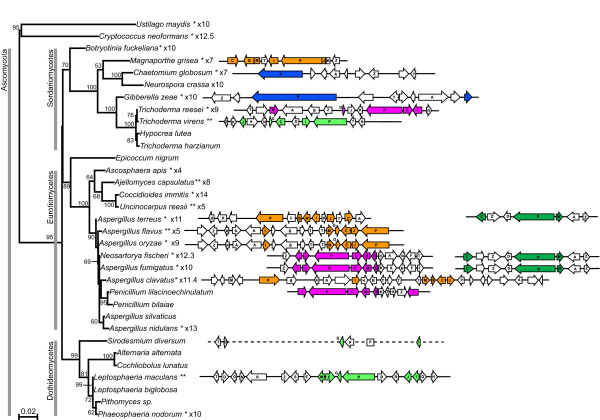
**Phylogenetic relationships between ascomycetes derived from 18S ribosomal DNA sequences showing the presence and subclade type of ETP-like gene clusters**. Numbers at nodes are bootstrap supports obtained from phyML. Cluster genes are coloured according to position within the phylogeny (identical colours to the branches in Fig. 3). The state of completeness of genome sequencing programs is shown where: * indicates that the genome is in assembly; ** indicates genome sequencing is in progress; xN indicates present coverage of the genome sequence, other information about the genome sequences is given in Additional file 1. Cluster details are as described in Fig. 2.

To compare the relationships of the cluster subclades to the organismal phylogenies of the ascomycete taxa where they occur, phylogenetic analyses using 18S ribosomal DNA as well as using the deduced amino acid sequence of heat shock protein 70 (hsp70) and beta-tubulin were performed. All three relationships were consistent and the phylogeny inferred from 18S ribosomal DNA sequences is shown in Fig. 5. The positions of taxa are also consistent with those previously reported [16]. When the presence of gene clusters was superimposed onto this organismal phylogeny, and the subclade identity indicated by colour, two points were obvious – firstly, the distribution of clusters in ascomycetes is discontinuous; and secondly, the relationship of cluster subclades does not always reflect the relationship of organisms (Fig. 5).

For some closely related species, one taxon had a cluster whilst the other taxon did not. Of two closely related dothideomycetes, *L. maculans *contains the sirodesmin PL cluster, whilst *P. nodorum *has no ETP-like clusters. Similarly, although several *Aspergilli *species have ETP-like clusters, *A. nidulans *does not (Fig. 5). The sordariomycete *C. globosum *has a cluster with three of the common genes, but the closely related species, *N. crassa *does not; indeed this latter fungus has very few secondary metabolite gene clusters [17].

The different subclades of ETP-like clusters were dispersed amongst the ascomycetes examined and did not simply correspond to taxonomic affinities (Fig. 5). The well-characterised *Aspergillus *genus in the eurotiomycete class contained a cluster from either subclade I or II, as well as a cluster from subclade IIIB. In three sordariomycete taxa, each had a different type of cluster (from subclade I, II or IIIA). Where some fungi had more than one cluster, these clusters did not occur in the same subclade. For instance *A. terreus *had a cluster with nearly all the common genes that grouped in subclade I and a second, smaller cluster (#2) that grouped in subclade IIIB. This second cluster was not found in the other species with subclade I clusters (*A. flavus*, *A. oryzae *and *A. clavatus*), however, it was present in *N. fischeri *and *A. fumigatus*, species that contained another cluster grouping in subclade II. Only one type of cluster (subclade IIIA) was characterised in the dothideomycetes (in *L. maculans*). However, the only genome sequence available for taxa of this class is that of *P. nodorum*.

### Synteny of genes within some clusters is conserved

The order and orientation of some genes within each cluster type was conserved. For example in the cluster from subclade I (orange) the order and orientation of *M, G, C, J *and *P *was conserved in *A. flavus, A. oryzae *and *A. clavatus*. Even the order of *M, G *and *C *in the more distantly related *M. grisea *was conserved, whereas in *A. terreus*, the order of these genes was not (Fig. 5). Similarly, the order of genes in the cluster from subclade II (pink) was conserved within the eurotiomycetes between *A. fumigatus *(gliotoxin gene cluster) and *N. fischeri *and for five genes in *P. lilacinoechinulatum*. However, of the cluster genes in subclade II basal member, *T. reesei*, only the order and relative direction of transcription of *P *and *C *were conserved. Amongst subclade IIIB, all clusters showed conserved gene synteny. Only two taxa, *L. maculans *and *T. virens *contained a cluster in IIIA subclade and gene order was not conserved in them.

### Gene replacement within clusters

In two cases, genes with similar putative function but different evolutionary origin were present in different clusters. For instance, each cluster had either an ABC transporter or a MFS transporter (Fig. 2). The gliotoxin gene cluster in *A. fumigatus *had a MFS transporter, as did clusters (also in subclade II) in *N. fischeri *and *P. lilacinoechinulatum*. However, the cluster in *T. reesei*, which is closely related and also in subclade II, had an ABC transporter. Similarly the sirodesmin PL cluster in *L. maculans *contained an ABC transporter, but the related subclade IIIA cluster in *T. virens *encoded an MFS transporter instead (Fig. 2). A second example of gene replacement is found with the dipeptidase (J) in the subclade I. This protein in *A. flavus*, *A. oryzae *and *A. clavatus *neither grouped with other subclade I proteins, nor even within the cluster clade, but rather with non-cluster paralogues from related *Aspergillus *spp. (Fig. 3).

## Discussion

### ETP clusters and their potential secondary metabolite products

This study has significantly expanded our knowledge of the distribution and composition of gene clusters resembling those known to synthesis ETP toxins. Clusters with most or all of the ten genes common to ETP clusters in *L. maculans *and *A. fumigatus *have been identified in nine additional ascomycete taxa. Also clusters comprising smaller numbers of these common genes along with other genes putatively involved in secondary metabolism have been identified, in some cases in taxa that contain complete ETP-like clusters. However, correlating these data with known ETP production is very challenging. Since only one ETP-like cluster with all genes predicted to be essential for gliotoxin production is present in two gliotoxin-producing fungi, *A. terreus *and *A. flavus*, this cluster is likely to be responsible for gliotoxin production. Interestingly these clusters belong to a different subclade to that responsible for gliotoxin production in *A. fumigatus*. *Aspergillus orzyae*, a fungus extensively used in the food industry and considered atoxigenic, has a cluster with extremely similar sequence and identical gene order to that of the putative gliotoxin gene cluster from *A. flavus*. However, this taxon is not known to produce gliotoxin, nor does it produce aflatoxins, despite an aflatoxin gene cluster [18]. Thus the presence of such secondary metabolite gene clusters does not always correlate with metabolite production. *Neosartorya fischeri*, which is also not reported to make any ETPs has a cluster with extremely similar sequence and identical gene order to that of the gliotoxin gene cluster from *A. fumigatus*. Until functional analyses are carried out with genes in all these clusters, the products (if any) of such clusters can only be speculated about. Functional (gene deletion) analyses have been carried out with the peptide synthetase in the ETP-like cluster in an isolate of *T. virens *that produces high levels of gliotoxin. The resultant mutant still produces gliotoxin indicating that this cluster is not responsible for gliotoxin production. The product (if any) of this cluster is unknown (Charles Kenerley, unpublished).

### Assembly and heredity of ascomycete ETP gene clusters

The monophyly of most of the cluster genes, and the fact that the closest relatives of the cluster genes clades are paralogues throughout the filamentous fungi, suggest that the cluster originated by recruitment of dispersed genes in the ascomycete genome. Given that most cluster genes represent genes for biochemical activities in multiple cellular processes, it is unsurprising that novel secondary metabolite pathways might have assembled from gene duplicates. This process of gene recruitment is reflected by with the replacement of the dipeptidase J by a non-cluster paralogue in the related taxa *A. flavus*, *A. oryzae *and *A. clavatus*. Such recruitment may also have occurred in the subclade IIIB clusters, where *A. fumigatus *and *N. fischeri *clusters contain a gene (with a FAD binding domain) that does not occur in the *A. terreus *IIIB cluster. This process most likely also accounts for the different types of transporters in the gene clusters. Amongst the clusters analysed in this study, only those from *G. zeae *and *C. globosum *are apparently derived independently.

The phylogeny of the modules of the peptide synthetase indicates that an early duplication of the ancestral gene occurred after the formation of the cluster, but prior to its dispersal in ascomycete lineages. This hypothesis is supported by previous studies on peptide synthetases in *Aspergillus *species [19]. Thus this character provides further support for the single heritage of cluster genes. Moreover, the consistent pattern of subclade relationships across the six cluster genes tested provides further evidence that the clusters have been inherited relatively intact, in several cases even with conserved gene synteny, although some changes to both gene content and gene order have since occurred. The few examples of non-cluster genes grouping within the cluster clades (e.g. peptide synthetase (P) from *T. reesei*; cytochrome P450 monooxygenase (C) from *A. flavus *and *N. fischeri*) may indicate relics of former clusters or cluster duplicates in these taxa that have since disassembled.

A similar mechanism of gene cluster assembly has been proposed for other secondary metabolite pathways in ascomycete fungi. Gene duplication, recruitment and then purifying selection has been proposed for the evolution of the aflatoxin and sterigmatocystin toxin gene clusters in the *Aspergillus *species [20]. These clusters contain about 25 genes, and evidence supporting gene duplication includes the presence of multiple gene homologues even in the same cluster. Recruitment of dispersed genes is also proposed for the evolution of a gene cluster involved in degradation of a nitrogen-containing molecule, allantoin (DAL) in *S. cerevisiae *[21].

A disjunct presence-absence distribution of secondary metabolite gene clusters, similar to that observed for the ETP clusters, has also been observed for other clusters in filamentous fungi. The dothidiomycete *Dothistroma septosporum*, has several linked genes with strong similarity to genes in the sterigmatocystin and aflatoxin gene clusters of the distantly related *Aspergillus *genus (eurotiomycetes). These *D. septosporum *genes encode enzymes that catalyse biosynthesis of a toxin, dothistromin, which has a similar structure to an intermediate in sterigmatocystin and aflatoxin biosynthesis [22].

Several theories have been proposed to account for the discontinuous distribution of gene clusters in fungi. Kroken et al. [23] have analysed the evolution of sequences for Type 1 polyketide synthetases, multifunctional enzymes that add two carbon molecules at a time to produce polyketides. Some polyketides are virulence factors in plant pathogens, or pigments such as melanin. Sequence analysis from a wide range of ascomycetes revealed eight polyketide synthase clades with discontinuous distributions. The authors proposed that this distribution can be best explained by gene duplication, divergence and gene loss, rather than by horizontal or lateral gene transfer. However, horizontal gene transfer has been postulated for the origin of the pea pathogenicity (PEP) gene cluster in the ascomycete, *Nectria haematococca *MPVI. Some genes in this cluster are involved in detoxifying a plant phytoalexin, pisatin [24]. This cluster is on a dispensable chromosome and comprises genes with no paralogues elsewhere in the genome and with a different G+C content and codon usage to flanking regions and to other chromosomes. This PEP cluster is present in the related *Fusarium oxysporum *f. sp.*pisi*, but is not in fungi that are closely related to *N. haematococca *MPVI, except for *N. boniensis *[24].

Not only are ETP-like clusters dispersed amongst some classes of ascomycetes and not others, but the phylogenetic affinities of the clusters are inconsistent with the phylogenetic affinities of their host organisms. Subclade IIIA is present in the sordariomycete, *T. virens*, and also in the dothidiomycete, *L. maculans*, but not in *P. nodorum*, which is the only the dothidiomycete whose complete genome sequence information is available. The eurotiomycetes, particularly the well-characterised *Aspergillus *species, contained two related types of clusters (subclades I and II) as well as the smaller cluster (subclade IIIB), which was related to the sirodesmin PL gene cluster. The sordariomycetes contained taxa with one of each of the clusters (subclades I, II and IIIA).

**Figure 6:**
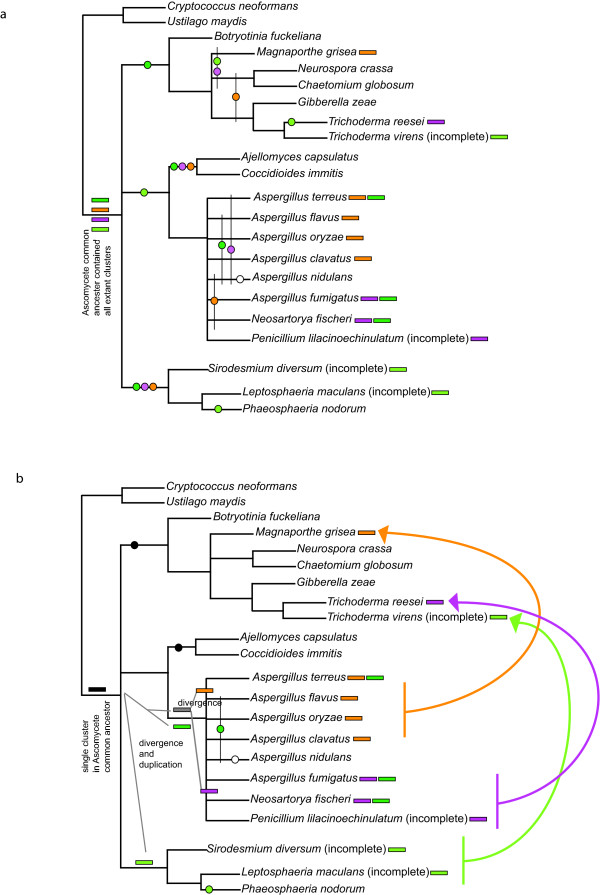
**Possible patterns of inheritance and loss of ETP-like clusters in ascomycetes**. Possible patterns of cluster inheritance and loss are mapped onto a conservative ascomycete phylogeny based on the 18S rDNA phylogeny (Fig. 5) with poorly supported nodes (<70 bootstraps) collapsed. Only taxa known to contain ETP clusters, or for which there is greater than X4 genomic sequence coverage are included to avoid falsely designating cluster loss events. Rectangles show presence and subclade type of cluster in a lineage (colours are as Fig. 3, black and grey indicate ancestral clusters). Circles indicate lineage-specific cluster loss events, where colours indicates subclade type loss, black indicates loss of all clusters, and open circle indicates loss of a cluster of unknown subclade type. Two possible scenarios are compared: (a) vertical inheritance only with the minimal number (17) of cluster losses; and (b) cluster divergence and spread by lateral transfer (arrows).

In view of the distribution, as well as the phylogenetic affinities of putative ETP clusters, two patterns of inheritance can be considered: 1) vertical inheritance and cluster loss alone; or 2) lateral transfer, vertical inheritance and cluster loss. From the phylogeny inferred by 18S rDNA, where nodes for which support is poor (less than 70 bootstraps) are collapsed, two possible patterns of inheritance can be mapped through filamentous fungi. With increased sampling and phylogenetic resolution the predicted number of losses will vary. However, with the current level of resolution and sampling, inheritance of clusters by vertical inheritance alone suggests that following cluster assembly in an ancestor of filamentous fungi, this cluster duplicated at least twice; once to generate subclade III (A and B), and then once more to give the sister subclades I and II (Fig. 6a). These multiple paralogous clusters must have all been inherited throughout ascomycetes, and multiple instances of cluster loss then ensued, some species losing all clusters and others retaining one or two different types. In this scenario, the most conservative estimate of minimal independent cluster loss events is 17 (using only taxa with greater than X4 sequence coverage, and phylogenetic relationships supported above a bootstrap cut-off of 70). However, with further sampling the predicted number of losses (shown as circles in Fig. 6a) is likely to be much higher. Some of these losses must also have occurred very recently within multiple closely related groups that now contain different cluster subclades (i.e. *Aspergillus *spp.). This scenario does not account for why some clusters have diverged relatively little despite presumably being inherited from the ancestor of ascomycetes. Additionally, although the number of taxa sampled to date is relatively few, no taxa with all major subclades have been found, even though three of these clades are all represented within a single genus (*Aspergillus*).

The second hypothesis is that following cluster assembly, these gene clusters diverged over time as ascomycetes diversified, creating the subclade relationships now observed (Fig. 6b). Later, entire gene clusters moved by horizontal gene transfer between ascomycete lineages, producing the discontinuous distribution and phylogenetic relationships seen in the proteins encoded by the six common genes tested here. This would imply multiple (at least three) instances of horizontal gene transfer. This scenario also includes cluster loss events, however, based on the conservative analysis as used for the first scenario with the same taxa, only five loss events are inferred (Fig 6b, circles). Combined with three independent lateral gene transfers, this second scenario provides a more parsimonious explanation of the data, although it still indicates a very complex pattern of gene cluster evolution in ascomycetes.

## Conclusion

Our results suggest that the epipolythiodioxopiperazines (ETP)-like gene clusters identified to date have a common origin, and have been inherited across ascomycetes relatively intact, rather than assembling independently in the different ascomycete lineages. The clusters in *Chaetomium globosum *and *Gibberella zeae *are exceptions, having apparently assembled independently, but their component genes are substantially different to those of the other clusters, and indeed these putative clusters may be implicated in some other metabolic function. The clade of putative ETP clusters is comprised of a small number of distinct subclades, which do not appear to always specify the particular toxins made, indicating ongoing refinement of the metabolic function of ETP clusters. Moreover, these clusters are represented in a discontinuous pattern across ascomycetes, and the phylogeny of the subclades does not reflect that of ascomycetes in which they occur. Although a mechanism describing this complex heredity cannot be conclusively identified, movement of entire clusters by horizontal gene transfer is the more parsimonious of these hypotheses to explain the discontinuous distribution of cluster types. The ability of fungi to transfer gene clusters that encode toxins or virulence factors presents important and novel implications, particularly for those fungi that are plant and animal pathogens.

## Methods

### Identification of ETP like clusters in genomes of filamentous fungi

Complete genome sequences for a range of ascomycetes and two basidiomycetes were downloaded from databases [see Additional file 1]. The presence of linked genes was sought by sequence similarity searches with genes in the sirodesmin PL and the gliotoxin gene clusters of *L. maculans *and *A. fumigatus*, respectively.

### Cloning and sequencing of ETP-like genes from *Sirodesmium diversum*

Fragments of *D, G, I, P *(module 1) and *T *were amplified from genomic DNA and cDNA of *S. diversum *using PCR with degenerate primers [see Additional file 2]. Primers were designed from multiple alignments of the genes of interest generated with ClustalW using the CODEHOP web interface [25]. Primers for the first module (mod 1) of *P *were chosen in regions conserved only between *sirP *(from *L. maculans*) and *gliP *(from *A. fumigatus*) and not other fungal peptide synthetase genes. The fragment for the second module (mod 2) was amplified with primers designed using the *L. maculans *genomic sequence. PCR amplification was carried out with 100 ng of genomic DNA and Invitrogen buffer, 4 mM MgCl_2_, 100 pmol each primer, 2.5 mM dNTPs and 1 U Invitrogen *Taq *polymerase. Annealing was at 94°C for 5 min followed by 5 cycles of Touchdown PCR (94°C for 15 sec, 60°C – 56°C for 15 sec,72°C for 90 sec) then 30 cycles (94°C for 15 sec, 55°C for 15 sec, 72°C for 90 sec) followed by 6 min at 72°C. Products were sequenced and outward facing primers designed and used in PCR amplification to join fragments of two sets of adjacent genes (*T *and *D*; *G *and *P*).

### Cloning and sequencing of ETP-like gene clusters from *Trichoderma virens *and from *Penicillium lilacinoechinulatum*

An isolate of a *Penicillium *species that producedgliotoxin was obtained from Dr Paul Waring, Australia. This isolate was originally named *P. terlikowskii *[11] but later identified and denoted as *Penicillium lilacinoechinulatum *Abe ex G. Smith (labstrain IBT 28164). This subsequent identification by Dr Jens Frisvad, Technical University of Denmark was on the basis of culture ex type, both micromorphologically, macromorphologically, physiologically and by its secondary metabolite patterns. A cosmid library of a gliotoxin-producing strain of *T. virens *(isolate G20-4VIB) was constructed in vector pMOCOSX and a cosmid library of *Penicillium lilacinoechinulatum *was constructed in the pWEB vector (Epicentre, USA). These libraries was probed with a 240 bp fragment amplified from genomic DNA of *T. virens *isolate G20-4VIB, and *P. lilacinoechinulatum*, respectively, using PCR and degenerate primers [see Additional file 2] designed from the *I *gene from *A. fumigatus *and *L. maculans*. Conditions for amplification were those used with the *S. diversum *gene fragments and intergenic regions.

Three different cosmids from the *T. virens *library hybridised; sequencing showed only one had genes expected to be in an ETP gene cluster (including the *I *homologue). One cosmid in the *P. lilacinoechinulatum *library hybridised to this probe. Preliminary sequence analysis showed that this cosmid had genes with best matches to *P, C *and *I*. These cosmids (30 kb inserts) were sequenced (X 2 coverage) by Macrogen (Seoul, South Korea). Genes were predicted using FGENESH [26] and predictions were confirmed with blastP searches. Of 14 genes predicted on the *T. virens *cosmid, 12 had good matches to genes in the *L. maculans *sirodesmin PL, and in the *A. fumigatus *gliotoxin biosynthetic gene clusters. On the 3' end of the cosmid, two genes with no sequence similarity to genes in ETP gene clusters were detected. The gene at the 5' end was a homologue of thetranscriptional regulator, *Z*, from *A. fumigatus*. Sequences were not available adjacent to this gene. Of 14 genes predicted on the *P. lilacinoechinulatum *cosmid, ten had good matches to genes on the sirodesmin PL and on the *A. fumigatus *gliotoxin gene clusters. These ten genes were flanked on the 3' end by a gene with no sequence similarity to genes in ETP clusters, and by three such genes at the 5' end, indicating that the cosmid contained a complete putative ETP gene cluster. The sequences of these two clusters are deposited in GenBank as accession EF429246 for *T. virens *and accession EF429247 for *P. lilacinoechinulatum*.

### Phylogenetic analyses of cluster genes

Sequences for eight of the ten common ETP cluster genes were obtained from GenBank, from the individual genome projects, and from the sequences of the clusters in *T. virens, S. diversum *and *P. lilacinoechinulatum*. These genes were non-ribosomal peptide synthetase (*P*), cytochrome P450 monooxygenases (*C*), glutathione *S*-transferase (*G*), ACCS (*I*), dipeptidase (*J*), methyl transferases (*M *and *N*), thioredoxin reductase (*T*). Sequences of the closest paralogues within the filamentous fungi and the closest orthologues from non-fungal taxa were also obtained. Paralogues were identified by BLAST from the genome projects and those paralogues that did not compromise the inclusion set of the cluster proteins, and therefore showed close relationships were analysed. Additionally sequences were collated for 18S ribosomal DNA, hsp-70 and beta-tubulin for all the fungi. Predicted protein alignments for protein-encoding genes were constructed using Clustal X [27] and refined in MacClade (Sinauer Associates, MA. USA). Alignments are available on request. Manual examination and BLAST results clearly indicated that non-fungal orthologues were much more distantly related and these were removed from the alignments to allow a greater character inclusion set for subsequent analyses. The alignments of T and N offered poor phylogenetic signal and accordingly the results were not analysed. After exclusion of characters that could not be aligned unambiguously, the matrix sizes (taxa × characters) for the individual protein analyses were I = 38 × 308; J = 30 × 276; M = 29 × 234; P = 36 × 306; C = 20 × 260; G = 36 × 133; Hsp 70 = 19 × 582; beta-tubulin = 23 × 362. The concatenated protein dataset combined these exclusion sets for the 13 cluster-containing taxa.

Maximum likelihood (ML) phylogenies were inferred using PhyML [28] with the Dayoff substitution matrix, eight categories of substitution rates. The alpha value and number of invariable sites were calculated from the datasets. The values obtained were I, a = 1.72, i = 0.0; J, a = 0.84, i = 0.0; M, a = 2.35, i = 0.01; P, a = 2.19, i = 0.06; C, a = 1.97, i = 0.02; G, a = 2.18, i = 0.0; hsp70, a = 0.28, i = 0.0; beta-tubulin; a = 0.3, i = 0.0. For distance analyses, gamma-corrected distances were calculated by TREE-PUZZLE 5.0 using the WAG substitution matrix with eight variable rate categories and invariable sites. Trees were inferred by weighted neighbor-joining using WEIGHBOR [29]. Bootstrap resampling was carried out using PUZZLEBOOT [30]. One hundred bootstrap trees were calculated with PhyML with the same parameters as the maximum likelihood tree but with four gamma corrections categories. Bootstraps were also calculated with WEIGHBOR. Alternative topologies for the concatenated dataset were tested using the Approximately Unbiased (AU) test [31]. Site likelihood values for test trees and 100 maximum likelihood bootstrap trees were calculated using TREE-PUZZLE 5.1 [32] and the parameters described above. AU tests were preformed using CONSEL version 0 [31].

The 18S ribosomal DNA nucleotide alignment gave a matrix size of 33 × 951. Maximum Likelihood phylogeny and ML bootstraps for 100 replicates were inferred using PhyML with the HKY method of substitution (transition/transversion ratio = 4.18) and nine categories of substitution rates with alpha (0.28) and the number of invariable sites (0.0) estimated from the dataset.

## Competing interests

The author(s) declares that there are no competing interests.

## Authors' contributions

All authors contributed to the concept of this manuscript. BJH and DMG conceived the study. AJC carried out the molecular studies, cluster identification and annotation. NJP and RFW made sequence alignments and phylogenetic analyses and interpretations. WN provided genome sequence data on several *Aspergillus *species and *Neosartorya fischeri*. DCS provided the *Trichoderma virens *cosmid library. BJH, NJP and RFW drafted the manuscript. All authors commented on drafts and approved the final manuscript.

**Table 1:** Genes in epipolythiodioxopiperazines (ETP) biosynthetic gene clusters

**Gene**	**Best match**
*P*	Non-ribosomal peptide synthetase
*T*	Thioredoxin reductase
*C*	Cytochrome P450 monooxygenase
*I*	Aminocyclopropane carboxylic acid synthase (ACCS)
*J*	Dipeptidase domain.
*M*	*O*-Methyl transferase domain.
*G*	Glutathione *S*-transferase domain.
*N*	Methyl transferase domain
*A*	Transporter
*Z*	Zinc binuclear cluster (Zn(II)_2_Cys_6_) regulator
*D*	Prenyl transferase
*O*	Aldehyde reductase
*E*	Cytochrome P450 monooxygenase
*S*	Progesterone 5-B reductase
*B*	Cytochrome P450 monooxygenase
*R*	Progesterone 5-B reductase
*Q*	Progesterone 5-B reductase
*K*	Hypothetical protein
*F*	Cytochrome P450 monooxygenase
*H*	Acetyl transferase

Cluster genes predicted in the biosynthesis in sirodesmin PL and gliotoxin in *Leptosphaeria maculans *and *Aspergillus fumigatus*, respectively (*P*, *T*, *C*, *I*, *J*, *M*, *G*, *N*,), as well as transporter (*A*) and transcriptional regulator (*Z*). Genes predicted to be involved in biosynthesis of side chains of these two ETPs are also listed (*D*, *O*, *E*, *S*, *B*, *R*, *Q*, *K*, *F*, *H*) [3, 4].

## Supplementary Material

Additional file 1 Details of complete genome sequences and gene clusters used in this study. Fungal species, isolate names, coverage of genome sequences and accession numbers of gene clusters are presented. Click here for file

Additional file 2 Primers used to amplify fragments of genes in ETP-like clusters in *Sirodesmium diversum*, *Trichoderma virens *or *Penicillium lilacinoechinulatum*. The sequences of primers used to amplify fragments of genes in ETP-like clusters in *Sirodesmium diversum*, *Trichoderma virens *or *Penicillium lilacinoechinulatum *are presented Click here for file

## References

[B1] Keller NP, Turner G, Bennett JW (2005). Fungal secondary metabolism - from biochemistry to genomics. Nat Rev Microbiol.

[B2] Gardiner DM, Waring P, Howlett BJ (2005). The epipolythiodioxopiperazine (ETP) class of fungal toxins: distribution, mode of action, functions and biosynthesis. Microbiol.

[B3] Gardiner DM, Cozijnsen AJ, Wilson LM, Pedras MS, Howlett BJ (2004). The sirodesmin biosynthetic gene cluster of the plant pathogenic fungus Leptosphaeria maculans. Mol Microbiol.

[B4] Gardiner DM, Howlett BJ (2005). Bioinformatic and expression analysis of the putative gliotoxin biosynthetic gene cluster of Aspergillus fumigatus. FEMS Microbiol Lett.

[B5] Balibar CJ, Walsh CT (2006). GliP, a multimodular nonribosomal peptide synthetase in Aspergillus fumigatus, makes the diketopiperazine scaffold of gliotoxin. Biochem.

[B6] Bok JW, Chung D, Balajee SA, Marr KA, Andes D, Nielsen KF, Frisvad JC, Kirby KA, Keller NP (2006). GliZ, a transcriptional regulator of gliotoxin biosynthesis, contributes to Aspergillus fumigatus virulence. Infect Imm.

[B7] Gardiner DM, Jarvis RS, Howlett BJ (2005). The ABC transporter gene in the sirodesmin biosynthetic gene cluster of Leptosphaeria maculans is not essential for sirodesmin production but facilitates self-protection. Fungal Genet Biol.

[B8] Cramer RA, Gamcsik MP, Brooking RM, Najvar LK, Kirkpatrick WR, Patterson TF, Balibar CJ, Graybill JR, Perfect JR, Abraham SN, Steinbach WJ (2006). Disruption of a nonribosomal peptide synthetase in Aspergillus fumigatus eliminates gliotoxin production. Eukaryot Cell.

[B9] Kupfahl C, Heinekamp T, Geginat G, Ruppert T, Hartl A, Hof H, Brakhage AA (2006). Deletion of the gliP gene of Aspergillus fumigatus results in loss of gliotoxin production but has no effect on virulence of the fungus in a low-dose mouse infection model. Mol Microbiol.

[B10] Wilhite SE, Lumsden RD, Straney DC (1994). Mutational analysis of gliotoxin production by the biocontrol fungus Gliocladium virens in relation to suppression of pythium damping-off.. Phytopathol.

[B11] Waring P, Eichner RD, Tiwari-Palni U, Mullbacher A (1987). Gliotoxin-E: a new biologically active epipolythiodioxopiperazine isolated from Penicillium terlikowskii.. Aus J Chem.

[B12] http://www.aspergillus.org.uk.

[B13] Pedras MSC, Seguin-Swartz G, Abrams SR (1990). Minor phytotoxins from the blackleg fungus Phoma lingam. Phytochem.

[B14] Curtis PJ, Greatbanks D, Hesp B, Cameron AF, Freer AA (1977). Sirodesmins A, B, C, and B, antiviral epipolythiopiperazine-2,5-diones of fungal origin: X-ray analysis of sirodesmin A diacetate. J Chem Soc.

[B15] Deng J, Carbone I, Dean RA (2007). The evolutionary history of cytochrome P450 genes in four filamentous ascomycetes. BMC Evolutionary Biology.

[B16] Berbee ML (2001). The phylogeny of plant and animal pathogens in the Ascomycota. Physiol Mol Plant Pathol.

[B17] Galagan JE, Calvo SE, Borkovich KA, Selker EU, Read ND, Jaffe D, FitzHugh W, Ma LJ, Smirnov S, Purcell S, Rehman B, Elkins T, Engels R, Wang S, Nielsen CB, Butler J, Endrizzi M, Qui D, Ianakiev P, Bell-Pedersen D, Nelson MA, Werner-Washburne M, Selitrennikoff CP, Kinsey JA, Braun EL, Zelter A, Schulte U, Kothe GO, Jedd G, Mewes W, Staben C, Marcotte E, Greenberg D, Roy A, Foley K, Naylor J, Stange-Thomann N, Barrett R, Gnerre S, Kamal M, Kamvysselis M, Mauceli E, Bielke C, Rudd S, Frishman D, Krystofova S, Rasmussen C, Metzenberg RL, Perkins DD, Kroken S, Cogoni C, Macino G, Catcheside D, Li W, Pratt RJ, Osmani SA, DeSouza CP, Glass L, Orbach MJ, Berglund JA, Voelker R, Yarden O, Plamann M, Seiler S, Dunlap J, Radford A, Aramayo R, Natvig DO, Alex LA, Mannhaupt G, Ebbole DJ, Freitag M, Paulsen I, Sachs MS, Lander ES, Nusbaum C, Birren B (2003). The genome sequence of the filamentous fungus Neurospora crassa. Nature.

[B18] van den Broek P, Pittet A, Hajjaj H (2001). Aflatoxin genes and the aflatoxigenic potential of Koji moulds. Appl Microbiol Biotechnol.

[B19] Cramer RA, Stajich JE, Yamanaka Y, Dietrich FS, Steinbach WJ, Perfect JR (2006). Phylogenomic analysis of non-ribosomal peptide synthetases in the genus Aspergillus. Gene.

[B20] Cary JW, Ehrlich KC (2006). Aflatoxigenicity in Aspergillus: molecular genetics, phylogenetic relationships and evolutionary implications. Mycopathologia.

[B21] Wong S, Wolfe KH (2005). Birth of a metabolic gene cluster in yeast by adaptive gene relocation. Nat Genet.

[B22] Bradshaw RE, Zhang S (2006). Biosynthesis of dothistromin. Mycopathologia.

[B23] Kroken S, Glass NL, Taylor JW, Yoder OC, Turgeon BG (2003). Phylogenomic analysis of type I polyketide synthase genes in pathogenic and saprobic ascomycetes. Proc Natl Acad Sci U S A.

[B24] Temporini ED, VanEtten HD (2004). An analysis of the phylogenetic distribution of the pea pathogenicity genes of Nectria haematococca MPVI supports the hypothesis of their origin by horizontal transfer and uncovers a potentially new pathogen of garden pea: Neocosmospora boniensis. Curr Genet.

[B25] Rose TM, Schultz ER, Henikoff JG, Pietrokovski S, McCallum CM, Henikoff S (1998). Consensus-degenerate hybrid oligonucleotide primers for amplification of distantly related sequences. Nucleic Acids Res.

[B26] FGENESH http://www.softberry.com.

[B27] Thompson JD, Gibson TJ, Plewniak F, Jeanmougin F, Higgins DG (1997). The CLUSTAL_X windows interface: flexible strategies for multiple sequence alignment aided by quality analysis tools. Nucleic Acids Res.

[B28] Guindon S, Gascuel O (2003). A simple, fast, and accurate algorithm to estimate large phylogenies by maximum likelihood. Syst Biol.

[B29] Bruno WJ, Socci ND, Halpern AL (2000). Weighted neighbor joining: a likelihood-based approach to distance-based phylogeny reconstruction. Mol Biol Evol.

[B30] Schmidt H, von Hasseler A (2003). Maximum-Likelihood Analysis Using TREE_PUZZLE.. Current Protocols in Bioinformatics.

[B31] Shimodaira H (2004). Approximately unbiased tests of regions using multistop-multiscale bootstrap resampling. Ann Stat.

[B32] Schmidt HA, Strimmer K, Vingon M, von Hasseler A (2002). TREE-PUZZLE: maximum likelihood phylogenetic analysis using quartets and parallel computing. Bioinformatics.

