# Supplementary material for: Origin and distribution of epipolythiodioxopiperazine (ETP) gene clusters in filamentous ascomycetes
Source: BMC Evol Biol. 2007 Sep 26;7:174. doi: 10.1186/1471-2148-7-174 (PMC2045112; doi:10.1186/1471-2148-7-174)
Supplement: Additional file 1 — Details of complete genome sequences and gene clusters used in this study. Fungal species, isolate names, coverage of genome sequences and accession numbers of gene clusters are presented. [file 1471-2148-7-174-S1.doc]

**Additional file 1. Details of gene clusters and complete genome sequences used in this study.**

* contigs were linked extensively by direct sequencing

| **Fungus** | **Isolate** | **Genome Coverage** | **Accession or contig #**  **of cluster** | **Sequencing agency** |
| --- | --- | --- | --- | --- |
| *Aspergillus clavatus* | NRRL 1 | X11.4 | NW_001517100 | TIGR |
| *Aspergillus flavus* | NRRL3357 | X5* | AAIH01000299 | TIGR |
| *Aspergillus fumigatus* | Af293 | X10* | AY838877.1; AAHF01000002 | TIGR |
| *Aspergillus oryzae* | RIB 40 | X9 | AP007154 | NITE |
| *Aspergillus terreus* | NIH2624 | X11 | NT_165927; NT_165935 | Broad |
| *Chaetomium globosum* | CBS 148.51 | X7 | supercontig # 8 | Broad |
| *Gibberella zeae* | G.z. PH-1 | X10 | supercontig # 1 | Broad |
| *Leptosphaeria maculans* | IBCN 18 | not sequenced | AY553235.1 |  |
| *Magnaporthe grisea* | 70-15 | X7 | AACU02000373 | Broad |
| *Neosartorya fischeri* | NRRL 181 | X12.3 | NZ_AAKE03000024;  NZ_AAKE03000007 | TIGR |
| *Penicillium lilacinoechinulatum* | IBT 28164 | not sequenced | EF429247 |  |
| *Sirodesmium diversum* | ATCC 36539 | not sequenced | AY571971-74; AHO13908 |  |
| *Trichoderma reesei* | QM9414 | X9 | scaffold #1 | JGI |
| *Trichoderma virens* | G20-4B | not sequenced | EF429246 |  |
| *Neurospora crassa* | OR74A | X10 | n/a | Broad |
| *Ascosphaera apis* | USDA-ARSEF7405 | X4 | n/a | Baylor College of Medicine |
| *Ajellomyces capsulatus* | G217B | X8 | n/a | Washington University |
| *Aspergillus nidulans* | FGSC A4 | X13 | n/a | Broad |
| *Botryotinia fuckleliana* | B05.10 | X10 | n/a | Broad |
| *Coccidioides immitis* | RS | X14 | n/a | Broad |
| *Phaeosphaeria nodorum* | SN15 | X10 | n/a | Broad |
| *Uncinocarpus reesii* | 1704 | X5 | n/a | Broad |
| *Cryptococcus neoformans* | JEC21 | X12.5 | n/a | TIGR |
| *Ustilago maydis* | 521 | X10 | n/a | Broad |
