# Supplementary material for: Origin and distribution of epipolythiodioxopiperazine (ETP) gene clusters in filamentous ascomycetes
Source: BMC Evol Biol. 2007 Sep 26;7:174. doi: 10.1186/1471-2148-7-174 (PMC2045112; doi:10.1186/1471-2148-7-174)
Supplement: Additional file 2 — Primers used to amplify fragments of genes in ETP-like clusters in Sirodesmium diversum, Trichoderma virens or Penicillium lilacinoechinulatum. The sequences of primers used to amplify fragments of genes in ETP-like clusters in Sirodesmium diversum, Trichoderma virens or Penicillium lilacinoechinulatum are presented [file 1471-2148-7-174-S2.doc]

**Additional file 2: Primers used to amplify fragments of genes in ETP-like clusters in *Sirodesmium diversum*, *Trichoderma virens* or *Penicillium lilacinoechinulatum***

| **Gene** | **Primer sequence** |
| --- | --- |
| *I* forward (f) | CCATCATGATGTGCAACCCNMAYAAYCC |
| *I* reverse (r) | GGATGCCGGTGCAGCMNADRTCYTT |
| *D* f | TGCGTCGTCCGATACACCWWHGARCCNAT |
| *D* r | TGCCGGTGCCGGANRTADATYTT |
| *P* mod1 f | CTACGGCCCCACCGARACNACNTG |
| *P* mod1 r | CGGCAGGTTGATCAGGAANCCNCKRTT |
| *P* mod 2f | TCACATGGTGAAATCGGCTA |
| *P* mod 2r | AATTCCCAACGCATCAACTC |
| *G* f | GTCAAGAACTGGATGACCTACCANACNGCNGC |
| *G* r | CGATGTCGGCGATGGTNGSNCKRTC |
| *T* f1 | TCATCGGCGCCGGNCCNGCNGG |
| *T* f2 | GGGCCGAAAGGTCGTCYTNGCNACNGG |
| *T* r | CCGCTGCTCGAAGCCRTSRCARAA |
